# Is Canada Moving towards a More Agile Regulatory Approval and Reimbursement Process with a Shifting Role for Real-World Evidence (RWE) for Oncology Drugs?

**DOI:** 10.3390/curroncol31090414

**Published:** 2024-09-18

**Authors:** Catherine Y. Lau, Nigel S. B. Rawson

**Affiliations:** 1 Independent Researcher, 158 Front Street E, Toronto, ON M5A 0K9, Canada; 2 Macdonald-Laurier Institute, Ottawa, ON K1N 7Z2, Canada; eastlakerg@gmail.com; 3 Canadian Health Policy Institute, Toronto, ON M5V 2Y5, Canada; 4 Centre for Health Policy Studies, Fraser Institute, Vancouver, BC V6J 3G7, Canada

**Keywords:** Health Canada, real-world evidence (RWE), Canada’s Drug Agency (cda-amc), previously CADTH, pCPA, NOC/c

## Abstract

Canada is known to have a complex pathway for new drug approval and reimbursement, resulting in delayed access for patients with serious and life-threatening diseases, such as cancer. Several recent publications from key stakeholders, including patients, physicians and policymakers, highlight patient helplessness, physician frustrations and policymakers entangled in a massive network of bureaucracy unable to make headway. Several quantitative and qualitative assessments using time from regulatory approvals to successful reimbursements confirm long review times and high rejection rates for oncology drugs, especially those receiving conditional approvals. A consensus forum of 18 Canadian oncology clinicians recently voiced frustration with the process and inability to deliver guideline-supported efficacious therapies to their patients. This manuscript compares data extracted from publicly available data sources from 2019 to June 2024 to previous publications. **Methods:** Public databases from Health Canada, the Canadian Agency for Drugs and Technologies in Health (CADTH), which is in the process of changing to Canada’s Drug Agency, and the pan-Canadian Pharmaceutical Alliance (pCPA) were reviewed and the data collected were analyzed with descriptive statistics. **Results:** From the data, three trends emerge, (i) an increasing number of oncology drugs are receiving conditional approvals from Health Canada, (ii) the percentage of conditionally approved oncology drugs receiving positive reimbursement recommendations from CADTH is still low but appears to be improving, but delays in access are now contingent upon pCPA deciding whether to negotiate price and then the duration of any negotiation, and (iii) real-world evidence is no longer part of the decision-making for conditional approvals. A slight increase in the positive endorsement of RWE used to support CADTH recommendations was observed. **Conclusions:** The lack of timely access to oncology drugs hurts Canadian patients. While a small trend of improvement appears to be emerging, longer-term data collection is required to ensure sustained patient benefits.

## 1. Introduction

The rapid evolution of targeted therapies for cancer patients in the past decade has put tremendous pressure on regulatory agencies globally to manage critical approval decisions based on early promising data rather than data confirmed by the gold-standard, randomized controlled trial (RCT) [1,2,3]. Novel small molecules, monoclonal antibodies and their antibody-drug-conjugates are powerful tools for targeting the proteins responsible for the mutagenesis leading to cancer. In the past decade, targeted therapies have proliferated, with now close to 40 for lung cancer and 20 for breast cancer [4,5]. Similar to chemotherapy regimens (often a cocktail of toxic and nonspecific chemicals), combinations of targeted therapies are delivering even more impressive results in cancer care [6].

Instead of being supportive, regulatory and reimbursement platforms have fallen short of aligning with the advancements in biology and innovative treatment strategies [7,8,9]. The US Food and Drug Administration (FDA) was the first to introduce regulatory reforms to accelerate reviews of targeted oncology therapies, including Real-Time Oncology Review, fast-track designation and breakthrough designation [10,11]. In May 2019, The FDA Oncology Center of Excellence (OCE) initiated Project Orbis to provide a framework for concurrent submission and collaborative reviews of oncology products among international partners. Since its inception, Project Orbis has received many market authorization applications and led to successful approvals of many oncology drugs for patients around the world [12].

Several recent publications documented significant delays for cancer patients in accessing these critical targeted therapies in Canada [7,8,13]. Unlike the FDA, which launched regulatory reforms to ensure accelerated decisions of new cancer treatments, Health Canada made minor modifications to one of its two existing pathways—Notice of Compliance with Conditions (NOC/c)—to approve new cancer therapies with promising clinical evidence, usually based on a small single-arm trial [1,2,13]. While NOC/c was originally designed to approve rare, life-threatening diseases with no treatment available, it has now been used for the onslaught of targeted oncology therapies, surpassing Notice of Compliance (NOC) approvals for the same drug category [13]. Until the condition is removed by a confirmatory study (phase III RCT), NOC/c approvals are deemed to carry a high level of uncertainty [14], which translates to a high percentage of negative reimbursement decisions from health technology assessment (HTA) agencies, including the Canadian Agency for Drugs and Technologies in Health (CADTH)—now renamed Canada’s Drug Agency (CDA)—and the Institut national excellence en santé et services sociaux (INESSS) (Quebec only).

The next step is the negotiation between manufacturers on the pricing and funding conducted between the government drug plans’ pan-Canadian Pharmaceutical Alliance (pCPA) and manufacturers based on a positive recommendation from CADTH or INESSS and a price deduction suggested by the HTA agency [15]. A negative recommendation denies further negotiation for most therapies. The high percentage of negative decisions, especially for NOC/c drugs, reported in several recent publications, is responsible for the significant delay in patient accessibility to treatment in Canada compared to many European countries with similar universal health system structures [16,17,18].

Canada’s complex approval and reimbursement landscape led to substantial delays in cancer patients’ access to appropriate treatments. A recent Targeted Literature Review (TLR) [7] reveals that important clinical endpoints, such as life years lost, overall survival and progression-free survival are most impacted by cancer patients’ delays in accessing new treatments. Physicians and patients are calling for system sea-changes to save the lives of cancer patients and prevent the deterioration of their quality of life [13].

This manuscript evaluates data from 2019 to June 2024 to assess any changes or modifications that might suggest different stakeholders are working towards a better solution for Canadian cancer patients.

## 2. Methods

The assessment targets were the New Active Substances (NAS) listed in the Health Canada submissions under review data completed between 2019 and 30 June 2024 [19,20]. Only antineoplastic drugs were recorded and reviewed (Supplementary Materials Table S1). The corresponding dates of submission to CADTH and dates of recommendations and dates of any pCPA negotiations or decisions not to negotiate were also captured.

The summary basis of decisions (SBD) or Regulatory Decision Summaries (RDS) for all listed products (Supplementary Materials Table S1) were reviewed [21] to categorize products into NOC, NOC/c or priority reviews (PRs). Section 3 of the SBD was reviewed to assess whether Project Orbis was part of the review process. Section 2 of the SBD (why was the drug approved) and Section 7 of the SBD (what are the scientific rationale of Health Canada’s decision) were examined to ascertain whether RWE was used for regulatory decisions based on search criteria with the keywords “real”, “historical”, “history”, “observation”, “natural”, “experience”, “registry”, “world” and “safety”, as used in a previous publication [22]. Five categories of RWE-use were created from the information derived to grade the RWD/E or historical data utilized by Health Canada for regulatory decision-making [22].

The times in calendar days of reviews by Health Canada, reviews by CADTH and negotiations with pCPA, and the time between CADTH recommendation and pCPA decision on whether to negotiate were calculated and analyzed with descriptive statistics.

RWE information was reviewed in CADTH reports according to the method described previously [23]. Briefly, the determination was made after a complete review of CADTH review reports that the assessment of fit-for-purpose RWE submitted by sponsors could be found in the clinical and pharmacoeconomic combined reports or the final clinical guidance reports. Section 1.2 (key results and interpretations) and Section 7 (supplemental questions) were reviewed in detail to understand how CADTH critically appraised the RWE information submitted by sponsors. CADTH’s comments in its clinical reviews on the parameters critical for the validity of RWE were extracted, and comparisons were made between positive and negative reimbursement recommendations (Supplementary Materials Table S2).

## 3. Results

### 3.1. Health Canada Review of Oncology Products: 2019 to June 2024

**Figure 1:**
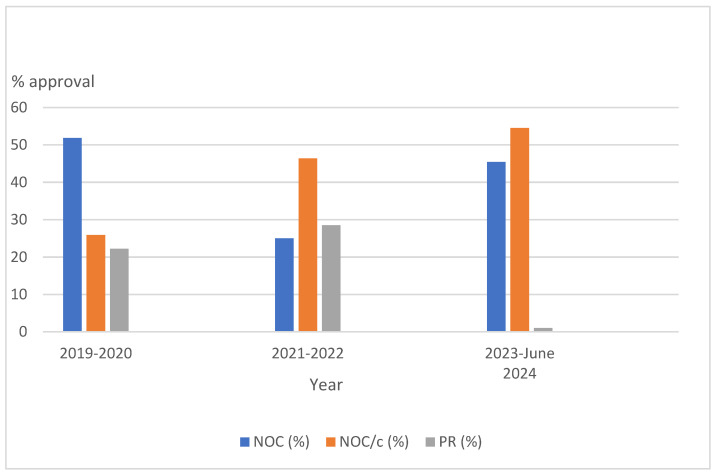
Approval status of oncology drugs approved from 2019 to June 2024. The analysis from 2023 to 30 June 2024 covers an eighteen months period.

Between 2019 and June 2024, 66 oncology drugs were approved by Health Canada. Table 1 lists the number of oncology drugs approved by year and by approval category. The total number of approvals peaked around 2019 and more products were approved between 2019 and 2021 than 2022 to June 2024. Figure 1 illustrates 2-year approval intervals, showing NOC approvals were consistent over the period with NOC/c approvals increasing rapidly and PR approvals diminishing drastically in 2023 to June 2024.

Health Canada joined the Project Orbis review initiative in 2019 [24]. The actual joint review process started around mid-year 2019, with the highest focus on NOC/c reviews. All but two NOC/c approved on or after 30 June 2021 were part of the Project Orbis review. The time used to review products under Project Orbis (median 277 days; IQ range 236–494 days) is similar to the average review time of NOC/c products from 2019 to June 2024 (median 279 days; IQ range 236–494 days) (Table 1 and Supplementary Materials Table S1).

The details of clinical reviews captured in Sections 2 and 7 were reviewed to search for the use of RWE in regulatory decision-making. Only seven reviews mentioned real-world evidence (Mylotarg, Enhertu, Tepmetko, Minjuvi, Pemazyre, Lumakras and Tecartus) (Supplementary Materials Table S1 and ref. [22]). All products approved using RWE were approved in 2020 and 2021. No RWE was mentioned in the review of products approved between 2022 to June 2024.

### 3.2. CADTH Review and pCPA Negotiations of Oncology Drugs Approved by Health Canada between 2019—June 2024

Of the 66 new oncology drugs that received approval from Health Canada between 2019 and the end of June 2024: 26 received an NOC, 26 an NOC/c and 14 had a priority review (Table 1).

All but 2 NOC drugs were submitted to CADTH, and 2 of the 24 submitted are currently being reviewed. Of the remaining 22 drugs, CADTH recommended 18 (81.8%) be reimbursed with or without conditions and/or criteria. No drug was reviewed within CADTH’s “typical timeline” of 180 calendar days, but 77.3% were reviewed within CADTH’s 190 business days (270 calendar days) target (Table 2). The pCPA’s decision regarding negotiation was made within its stated target of 40 business days (60 calendar days) or less following the reimbursement recommendation for only 22.7% of the drugs. Price negotiations between the pCPA and the drug developer were ongoing for 3 of the 22 drugs. The pCPA decided not to pursue a negotiation for 3 of the other 19 medicines, all of which received a negative recommendation from CADTH. Negotiations were completed within the pCPA’s stated target of 90 business days (130 calendar days) or less for 37.5% of the drugs.

Two of the twenty-six NOC/c drugs were not submitted to CADTH and two were currently being reviewed. Twelve of the twenty-two drugs (54.5%) with a completed CADTH review received a positive reimbursement recommendation. Few reviews (13.6%) were completed within CADTH’s “typical timeline”, while 59.1% were completed within 270 days. One drug (Epkinly) was reviewed under CADTH’s new time-limited reimbursement recommendation process for NOC/c drugs [25], which was completed in 199 days—still longer than the claimed “typical timeline”. The pCPA’s decision about whether to negotiate was within its target time for only 11.1% of the NOC/c drugs and completed negotiations were within its target for 54.5% of the negotiated drugs. Remarkably, the pCPA engaged with Epkinly’s developer before CADTH’s recommendation was issued, which it has not done previously.

All the oncology drugs with a priority review were evaluated by CADTH, with 78.6% being reviewed within 270 days; none were reviewed within the agency’s 180-day “typical timeline”. One of the priority review drugs was under consideration for negotiation by the pCPA. The decision regarding negotiation was made within the pCPA’s target for only 7.7% of the medicines. Two negotiations were currently in progress, while 36.4% of the completed negotiations were accomplished within the pCPA’s target for negotiations.

For products approved by Health Canada between 2019 and 2021, 76.7% with completed CADTH reviews were given a positive reimbursement recommendation, while 100% of products approved in 2022 and 2023 received a positive reimbursement recommendation. For NOC/c approvals, 37.5% and 100% of products received a positive reimbursement recommendation, respectively.

The sponsors of nine drugs (Piqray, Abecma, Tepmetko, Minjuvi, Pemazyre, Zepzelca, Lumakras, Jemperli and Rybrevant) requested a reconsideration of CADTH’s recommendation for major revisions, and a procedural review was also requested for Minjuvi and Pemazyre. All but Rybrevant received a final negative recommendation. Reconsideration was requested for major revisions for Rylaze by government drug plans. The impact on the time required for reimbursement reviews due to reconsiderations and procedural reviews is unknown, because the extent of any delay is not recorded by CADTH. A re-analysis excluding these drugs did not demonstrate a significant change in the overall results. It is also unknown whether the reconsideration for Rybrevant changed its recommendation from negative to positive. However, based on the final recommendations for the other seven drugs being negative, it appears that reconsiderations and procedural reviews rarely change CADTH’s recommendations.

All oncology drugs that received a positive reimbursement review had a pCPA price negotiation and 82% of the negotiations were successful. In contrast, only three (30%) of the drugs with a negative reimbursement recommendation were negotiated and only two were successful (Supplementary Materials Table S1).

The use of RWE in CADTH recommendations for NOC/c products approved by Health Canada from 2019 to June 2024 not included in a previous publication [23] was reviewed (Supplementary Materials Table S2). Four products, Gavreto, Carvykti, Tecvayli and Elrexfio (approved in 2023), with positive reimbursement recommendations, received a moderately positive review mentioning that the RWE data were generally consistent with the expectations of clinical experts or better than comparators. Products with recommendations of non-reimbursement all received negative reviews of the RWE, and two with positive recommendations (Columvi and Epkinly) (Supplementary Materials Table S2) received negative RWE reviews.

## 4. Discussion

The availability of new medications to patients is a long and arduous process in Canada. In a recent speech, Doug Ford, premier of Ontario, summarized it succinctly: “Currently, patients in Canada wait almost two years to access life-saving breakthrough medicines, a year longer than in other developed countries, placing us last in the G7” [26]. For this message to reach the level of a premier who boldly announced that one of his focuses as the chair of the Council of Federation is to ensure Canadian patients have the same timely access to life-saving treatments as patients in the rest of the world is monumental, if this recognition results in positive change. In recent years, evidence supporting delayed access to new and life-saving medications for Canadian patients has become stronger and louder, as witnessed by the rising number of peer-reviewed publications by Canadian and international authors [16,27].

Canadian regulatory bodies, HTA organizations and reimbursement agencies are aware of the issues but are deterred by fear that high drug prices could bankrupt the drug budget. These agencies are open to discussion regarding ways to improve drug access. However, resource constraints, especially from review divisions, layers of uncertainties with data packages, economic evaluations and value for money, hold back decisions to make new and life-prolonging medications available to Canadian patients [27,28].

The arrival of a tsunami of targeted therapies offering potent efficacies against cancer and with better safety profiles is not unexpected. The FDA realized that waiting for definitive results, such as overall survival or progression-free survival from multi-year-long randomized clinical studies, deprive cancer patients of life-saving treatments. The FDA will approve targeted oncology drugs with novel mechanisms of action after a single-arm trial and, in some cases, supported by historical data or RWE in comparable patient populations [29]. Health Canada decided to use the existing NOC/c pathway to provide access to these promising new therapies. Confirmatory studies aligned with the indications have to be ongoing for this conditional approval to be granted [2,14]. However, the prolonged fulfillment of NOC/c conditions could potentially expose patients to drugs with faster approvals based on the promising evidence of efficacy, that are less effective or have a higher risk than existing standard-of-care therapies. A recent analysis of NOC/c approvals by Martin et al. indicated that only 7 out of 92 NOC/c oncology drugs approved between 1998 and 2021 had been removed, withdrawn, or suspended. Moreover, 82.4% of the drugs issued NOC/c between 2018 and 2021 were integrated into standard-of-care practices [2]. The authors hence concluded that the downsides of earlier approvals are few.

Health Canada also issued two guidance policies for the use of RWE in regulatory decision-making in April 2019 [30,31], focusing on requirements for regulatory approvals. A study conducted to evaluate the use of RWE by Health Canada in the approval of oncology and rare disease drugs showed that Health Canada used much less RWE in regulatory decision-making than the FDA and EMEA [22] for approvals between 2020 and 2021. Of the 29 oncology drugs approved, only 7 (2 in 2020 and 5 in 2021) mentioned RWE. The current study evaluated the use of RWE in oncology drug approvals from 2019 to June 2024 and compared to the previous publication [22], only one more drug approved in 2021 was reviewed using RWE historical data. None of the drugs approved between 2022 to June 2024 mentioned the use of RWE as part of the regulatory decision-making. This can potentially put Health Canada out of step with the FDA, which has been incorporating more RWE into regulatory decision-making, especially for validating whether an “external control arm” can serve as the comparator arm of a single-arm trial. The FDA is exploring whether using a well-validated external control arm with the treatment arm could yield results comparable to RCTs [29].

Health Canada has been endorsing and collaborating with CADTH on RWE initiatives [32]. CADTH was active both in revising the RWE guidance document and engaging stakeholders in panel discussions on how to incorporate RWE into CADTH submissions. The guidance document finalized in 2023 [33] had a long stakeholder consultation period, suggesting that CADTH is actively interested in improving the review of RWE submitted to them. The current study also evaluated CADTH review reports for the use of RWE and found that instead of providing the same negative reviews of RWE for all drugs regardless of the recommendations [23], at least four drugs reviewed between 2022 and 2023 received positive appraisals regarding the provided RWE information. Although some drugs with positive recommendations still received negative comments on submitted RWE, this is a step in the right direction (Supplementary Materials Table S2). More recently, CADTH published a report from its time-limited Industry Task Force including a summary report from the Post-Marketing Drug Evaluation (PMDE) committee, a multi-stakeholder panel advising on the best collaborative approach for industry and HTA to incorporate RWE into PMDE [34]. Several feasibility initiatives will be launched, and longer-term data will be needed to assess the value of these programs.

For oncology products approved between 2019 to June 2024, review times by CADTH and pCPA and time to start negotiation by pCPA showed inadequate alignment with the agencies’ stated targets. While some delays could originate from the sponsors, online accessible times in review are not detailed enough to reveal where the delays took place. Sponsors of several drugs requested CADTH’s reconsideration of negative decisions. It is not clear whether reconsiderations impacted CADTH timelines, but a reanalysis of CADTH review times did not show significant differences with or without these products. The time required for Epkinly, which received a positive recommendation under the CADTH new time-limited reimbursement pathway, took 199 days, closer to the target of 180 days, and if sustainable and governments list the drugs quickly, it will enable cancer patients to receive their treatments promptly.

## 5. Conclusions

While the availability of new treatments to cancer patients is still a challenge in Canada, promoting collaboration among key stakeholders, revising current regulatory platforms and encouraging openness to accepting new data formats will be critical parameters for determining the long-term benefits of cancer patients.

## Figures and Tables

**Table 1:** Approvals of oncology drugs by Health Canada 2019—30 June 2024.

Years	2019	2020	2021	2022	2023	2024 ^
**NOC**	8	6	1 #	6 *	4	1
**NOC/c**	5	2	11 **	2 **	5 **	1 **
**PR**	3	3	5 ***	3	0	0
**Total**	16	11	17	11	9	2

^ Only data up to June 30 were analyzed. Project Orbis: NOC * 1 out of 6 in 2022, NOC/c ** 7 out of 11 in 2021 (after June 30), 2 out of 2 in 2022, 3 out of 5 in 2023, 1 out of 1 in 2024, PR *** 2 out of 5 in 2021; # two products Mektovi and Braftovi were approved as separate products but used in combination for CADTH and pCPA considerations; NOC (Notice of Compliance), NOC/c (Notice of Compliance with Conditions), PR (priority review).

**Table 2:** Summary of time taken for CADTH reviews, pCPA decision about negotiation, and pCPA negotiation.

Regulatory Status		CADTH Review	pCPA Decision	pCPA Negotiation
NOC	Number	22	22	16
	Median	234 days	92 days	141 days
	IQ range	223–255 days	64–184 days	90–205 days
	Within relevant target *	77.3%	22.7%	37.5%
NOC/c	Number	22	18	11
	Median	235 days	125 days	110 days
	IQ range	200–319 days	91–204 days	86–142 days
	Within relevant target	59.1%	11.1%	54.5%
Priority review	Number	14	13	11
	Median	234 days	147 days	167 days
	IQ range	214–255 days	108–182 days	107–264 days
	Within relevant target	78.6%	7.7%	36.4%

IQ: Inter-quartile; * Relevant targets are 270 days for CADTH review, 60 days for pCPA decision, and 130 days for pCPA negotiation.

## Data Availability

The original contributions presented in the study are included in the article; further inquiries can be directed to the corresponding author.

## Supplementary Materials

The following supporting information can be downloaded at: https://www.mdpi.com/article/10.3390/curroncol31090414/s1, Table S1: Submission and Review Completion dates by Health Canada, CADTH and pCPA for Oncology Drugs; Table S2: Use of RWE in CADTH completed reviews for NOC/c from 2019-June 2024 not reviewed previously [23].
