# Supplementary material for: Is Canada Moving towards a More Agile Regulatory Approval and Reimbursement Process with a Shifting Role for Real-World Evidence (RWE) for Oncology Drugs?
Source: Curr Oncol. 2024 Sep 18;31(9):5599–607. doi: 10.3390/curroncol31090414 (PMC11430900; doi:10.3390/curroncol31090414)
Supplement: Supplementary file 1 [file curroncol-31-00414-s001.zip › curroncol-3197335-supplementary.pdf]

**Supplemental Table S1.** Submission and Review Completion dates by Health Canada, CADTH and pCPA for Oncology Drugs.

| Drugs (generic name) | Health Canada | | CADTH | | | pCPA | | |
|---|---|---|---|---|---|---|---|---|
| | Submission date | Approval date | Submission date | Completion date | Recommendation | Outcome | Decision to negotiate | Negotiation ended |
| **Notice of Compliance (NOC)** | | | | | | | | |
| Dacogen (decitabine) | 2018-06-28 | 2019-07-11 | 2020-10-09 | 2021-09-22 | Reimburse (C) | No agreement | 2022-02-16 | 2022-04-22 |
| Vizimpro (dacomitinib) | 2018-03-16 | 2019-02-26 | 2018-09-18 | 2019-05-31 | Reimburse (C) | No agreement | 2019-08-13 | 2020-02-11 |
| Verzenio (abemacicib) | 2018-04-06 | 2019-04-05 | 2018-12-03 | 2019-07-05 | Reimburse (C) | No agreement | 2019-09-30 | 2020-04-09 |
| Talzenna (talazoparib) | 2018-09-28 | 2020-09-30 | Not filed | | | | | |
| Zejula (niraparib) | 2018-05-31 | 2019-06-27 | 2020-09-21 | 2021-04-29 | Reimburse (C) | LOI | 2021-06-01 | 2021-08-11 |
| Decitabine | 2018-06-28 | 2019-07-11 | Not filed | | | | | |
| Nerlynx (neratinib) | 2018-06-21 | 2019-07-16 | 2019-04-18 | 2019-12-05 | Do not reimburse | Not pursued | | 2020-02-04 |
| Calquence (acalabrutinib) | 2018-03-15 | 2019-08-23 | 2020-04-07 | 2020-11-17 | Reimburse (C) | LOI | 2021-05-27 | 2021-10-05 |
| Mylotarg (gemtuzumab ozogamicin) | 2018-12-19 | 2019-11-28 | 2019-08-09 | 2020-04-02 | Reimburse | LOI | 2020-06-17 | 2020-08-12 |
| Nubeqa (darolutamide) | 2019-03-27 | 2020-02-20 | 2019-08-27 | 2020-04-22 | Reimburse (C) | LOI | 2020-10-23 | 2021-02-18 |
| Piqray (alpelisib) | 2019-04-17 | 2020-03-11 | 2021-04-21 | 2022-01-26 | Do not reimburse | No agreement | 2022-12-16 | 2023-03-14 |
| Daurismo (glasdegib) | 2019-03-15 | 2020-04-28 | 2020-05-06 | 2021-01-08 | Do not reimburse | Not pursued | | 2021-02-23 |
| Sarclisa (isatuximab) | 2019-06-28 | 2020-04-29 | 2020-08-17 | 2021-04-01 | Reimburse (C) | LOI | 2022-05-13 | 2023-03-07 |
| Odomzo (sonidegib) | 2019-07-04 | 2020-06-12 | 2020-06-19 | 2021-04-29 | Do not reimburse | Not pursued | | 2021-06-04 |
| Inrebic (fedratinib) | 2019-07-19 | 2020-07-27 | 2020-11-05 | 2021-06-21 | Reimburse (C) | LOI | 2021-12-21 | 2022-05-13 |
| Mektovi (binimetinib)/Braftovi(encorafenib) | 2020-03-20 | 2021-03-02 | 2020-12-16 | 2021-07-26 | Reimburse (C) | LOI | 2021-11-03 | 2022-07-06 |
| Breyanzi (isocabtagene maralecucel | 2020-12-17 | 2022-05-06 | 2021-08-09 | 2022-06-29 | Reimburse (C) | LOI | 2023-01-20 | 2023-12-12 |
| Xpovio (selinexor) | 2021-05-28 | 2022-05-31 | 2022-01-14 | 2022-07-29 | Reimburse | LOI | 2022-12-20 | 2023-05-15 |
| Scemblix (asciminib) | 2021-08-13 | 2022-06-21 | 2022-01-20 | 2022-08-05 | Reimburse (C) | LOI | 2022-10-21 | 2023-03-09 |
| Koselugo (selumetinib) | 2020-09-09 | 2022-08-31 | 2022-10-28 | 2023-05-29 | Reimburse (C) | Active | 2024-03-12 | |
| Rylaze (crisantaspase, recombinant) | 2021-09-27 | 2022-09-02 | 2022-08-18 | 2023-04-23 | Reimburse (C) | LOI | 2023-06-02 | 2023-07-04 |
| Welireg (belzutifan)* | 2021-07-09 | 2022-07-11 | 2023-01-23 | 2023-09-01 | Reimburse (C) | Active | 2023-12-07 | |
| Imjudo (durvalumab+ tremelimumab) | 2022-03-24 | 2023-08-31 | 2022-12-15 | 2023-11-03 | Reimburse (C) | LOI | 2023-12-08 | 2024-02-28 |
| Opdualag (nivolumab+ relatimab) | 2022-11-03 | 2023-10-10 | 2023-07-10 | 2024-02-21 | Reimburse (C) | Active | 2024-06-21 | |
| Orgovyx (relugolix) | 2022-11-03 | 2023-10-10 | 2023-11-14 | | Active | | | |
| Asparlas (calaspargase pegol) | 2022-09-29 | 2023-11-08 | 2023-05-12 | 2024-01-22 | Reimburse (C) | LOI | 2024-03-22 | 2024-06-21 |
| Truqap (capivasertib) | 2023-04-28 | 2024-01-24 | 2024-01-14 | Active | | | | |
| **Notice of Compliance with Conditions (NOCc)** | | | | | | | | |
| Idhifa (enasidenib) | 2018-06-15 | 2019-02-06 | 2019-04-19 | 2019-10-31 | Do not reimburse | Not pursued | | 2023-06-29 |
| Lorbrena (lorlatinib) | 2018-04-26 | 2019-02-22 | 2019-11-06 | 2020-01-30 | Do not reimburse | Not pursued | | 2020-04-21 |
| Libtayo (cemiplimab) | 2018-07-27 | 2019-04-10 | 2019-07-09 | 2020-01-22 | Reimburse (C) | LOI | 2020-06-08 | 2020-11-04 |
| Vitrakvi (larotrecinib) | 2018-09-18 | 2019-07-10 | 2019-02-25 | 2019-10-31 | Do not reimburse | Not pursued | | 2019-12-30 |

| | | | | | | | | |
|---|---|---|---|---|---|---|---|---|
| Balversa (erdafitinib) | 2019-02-08 | 2019-10-25 | 2024-02-14 | Active | | | | |
| Rozlytrek (entrectinib) | 2019-05-07 | 2020-02-10 | 2020-01-08 | 2021-01-27 | Reimburse (C) | LOI | 2021-05-04 | 2021-07-06 |
| Polivy (polatuzumab vedotin) | 2019-10-04 | 2020-07-09 | 2020-09-29 | 2021-04-21 | Reimburse (C) | LOI | 2021-07-21 | 2021-12-01 |
| Enhertu (trastuzumab deruxtecan) | 2020-07-24 | 2021-04-15 | 2022-03-23 | 2022-09-28 | Reimburse (C) | LOI | 2022-12-21 | 2023-05-29 |
| Abecma (idecabtagene vicleucel) | 2020-09-17 | 2021-05-26 | 2020-12-16 | 2021-11-12 | Do not reimburse | Not pursued | | 2024-04-18 |
| Tepmetko (tepotinib) | 2020-07-31 | 2021-05-27 | 2021-08-30 | 2022-08-24 | Do not reimburse | LOI | 2023-03-09 | 2023-06-22 |
| Retevma (selpercatinib) | 2020-09-10 | 2021-06-15 | 2021-08-17 | 2022-04-28 | Reimburse (C) | LOI | 2022-11-17 | 2023-03-31 |
| Gavreto (pralsetinib)* | 2020-08-09 | 2021-06-30 | 2022-09-03 | 2022-10-18 | Reimburse (C) | No agreement | 2023-06-23 | 2023-07-24 |
| Minjuvi (tafasitamab)* | 2020-04-12 | 2021-08-19 | 2021-11-19 | 2022-09-29 | Do not reimburse | LOI | 2023-05-26 | 2023-08-08 |
| Pemazyre (pemigatinib)* | 2020-09-17 | 2021-09-17 | 2021-06-23 | 2022-04-08 | Do not reimburse | Not pursued | | 2022-07-29 |
| Truqap (capivasertib)* | 2023-04-28 | 2024-01-24 | 2024-02-14 | | Active | | | |
| Truseltiq (infigratinib)* | 2020-11-30 | 2021-09-27 | Not filed | | | | | |
| Zepzelca (lurbinectedin)* | 2020-12-16 | 2021-09-29 | 2022-02-24 | 2023-01-12 | Do not reimburse | Not pursued | | 2023-08-04 |
| Lumakras (sotorasib)* | 2021-01-14 | 2021-10-22 | 2022-08-19 | 2024-03-19 | Do not reimburse | Under consideration | | |
| Jemperli (dostarlimab)* | 2021-03-26 | 2021-12-23 | 2021-10-07 | 2022-08-24 | Do not reimburse | Not pursued | | 2022-10-19 |
| Tabrecta (capmatinib)* | 2021-08-19 | 2022-05-06 | Not filed | | | | | |
| Carvykti (citacabtagene autoleucel) | 2022-03-04 | 2023-02-09 | 2022-09-23 | 2023-05-01 | Reimburse (C) | Active | 2023-11-10 | |
| Columvi (glofitamab) | 2022-06-23 | 2023-03-24 | 2023-07-18 | 2024-01-02 | Reimburse (C) | LOI | 2024-04-03 | 2024-07-22 |
| Rybrevant (amivantamab)* | 2021-07-30 | 2023-03-30 | 2022-04-21 | 2023-03-01 | Reimburse (C) | No agreement | 2023-06-01 | 2023-11-03 |
| Tecvayli (teclistamab)* | 2022-11-02 | 2023-09-23 | 2023-08-31 | 2024-04-08 | Reimburse (C) | Under consideration | 2024-06-30 | |
| Epkinly (epcoritamab)* | 2023-01-12 | 2023-10-13 | 2023-11-14 | 2024-05-31 | Reimburse (C) | LOI (pTAP negotiation) | 2024-04-12 | 2024-07-19 |
| Elrexfio (elranatamab)* | 2023-03-21 | 2023-12-06 | 2023-11-09 | 2024-05-31 | Reimburse (C) | No record | | |
| Talvey (talquetamab) * | 2023-08-15 | 2024-04-30 | 2024-05-01 | 2024- | Active | | | |
| **Priority Review** | | | | | | | | |
| Lutathera (lutetium Lu177 dotatate) | 2018-06-18 | 2019-01-09 | 2018-07-30 | 2019-08-01 | Reimburse (C) | No agreement | 2019-09-26 | 2019-12-30 |
| Yescarta (axicabtagene ciloleucel) | 2018-07-19 | 2019-02-13 | 2022-06-09 | 2023-02-03 | Reimburse (C) | Active | 2023-09-26 | |
| Xospata (giltertinib) | 2019-05-15 | 2019-12-23 | 2019-10-19 | 2020-05-20 | Reimburse (C) | LOI | 2020-08-17 | 2021-03-15 |
| Tukysa (tucatinib) | 2020-01-20 | 2020-06-05 | 2021-03-26 | 2021-11-17 | Reimburse (C) | LOI | 2022-05-18 | 2022-09-14 |
| Qinlock (ripretinib) | 2019-12-23 | 2020-06-19 | 2021-10-15 | 2022-04-28 | Reimburse (C) | LOI | 2022-07-12 | 2023-05-12 |
| Inqovi (decitabine+ cedazuridine) | 2019-12-31 | 2020-07-07 | 2020-10-09 | 2021-09-22 | Reimburse (C) | No agreement | 2022-02-16 | 2022-04-22 |
| Brukinsa (zanubrutinib) | 2020-08-13 | 2021-03-01 | 2021-05-21 | 2021-12-21 | Reimburse (C) | LOI | 2022-10-05 | 2022-12-22 |
| Tecartus (brexucabtagene autoleucel) | 2020-11-13 | 2021-06-08 | 2020-12-18 | 2021-08-06 | Reimburse (C) | LOI | 2021-11-22 | 2022-11-10 |
| Trecondyv (treosulfan) | 2020-09-21 | 2021-06-25 | 2023-07-14 | 2024-02-28 | Reimburse (C) | Under consideration | | |
| Trodelvy (sacituzumab govitecan) | 2021-01-25 | 2021-09-24 | 2021-06-30 | 2022-01-22 | Reimburse (C) | LOI | 2022-07-29 | 2023-03-09 |

| | | | | | | | | |
|---|---|---|---|---|---|---|---|---|
| Padcev (enfortumab)* | 2021-06-04 | 2021-10-29 | 2021-06-23 | 2022-01-06 | Reimburse (C) | LOI | 2022-06-28 | 2022-11-28 |
| Pluvicto (lutetium vipivotide tetraxetan) | 2022-01-28 | 2022-08-25 | 2022-07-21 | 2023-03-22 | Reimburse (C) | Active | 2023-08-29 | |
| Poteligeo (mogamulizumab) | 2021-03-21 | 2022-06-02 | 2021-09-13 | 2022-08-12 | Reimburse (C) | LOI | 2022-12-19 | 2024-03-13 |
| Kimmtrak (tebentafsup)* | 2021-11-19 | 2022-06-21 | 2022-04-21 | 2023-01-04 | Reimburse (C) | LOI | 2023-05-09 | 2023-10-23 |

LOI: Letter of intent; Reimburse (C): Reimburse with conditions and/or criteria; pTAP: pCPA Temporary Access Process

Times in review by Health Canada, CADTH, time to negotiate and negotiation period by pCPA: Calculated using dates listed above

* Reviewed in Orbis;

**Supplemental Table S2:** Use of RWE in CADTH completed reviews for NOC/c from 2019-June 2024 not reviewed previously [23]

| **Names of NOC/c products /Health Canada approval data**<br>**CADTH recommendations**<br>**RC = Recommend with Conditions**<br>**DNR = Do not Reimburse** | **Critical appraisals of RWE data with uncertainties** | **Critical appraisals of RWE data with positive trends** |
|---|---|---|
| Gavreto (pralsetinib)<br>2021-06-31<br>RC | | With these limitations in mind, it should also be noted that all results were directionally consistent and in line with the clinical expert's expectations that pralsetinib is likely a better option for patients than the comparators included in the ITC analysis…... *The results found patients receiving pralsetinib showed a statistically significant benefit in OS and PFS compared with the comparators chosen, which is consistent with the expectations of the clinical expert consulted*; however, the same limitations are present as in the sponsor-submitted ITC. |
| Minjuvi (tafasitamab)<br>2021-08-19<br>DNR | The sponsor submitted 3 indirect treatment comparisons (ITCs) that compared patients in the L-MIND study to patients treated with other therapies. However, given the methodological limitations of the analyses (i.e., heterogeneity, matching based on a limited number of variables, and small sample sizes), pERC was unable to determine the comparative efficacy of tafasitamab plus lenalidomide relative to other therapies | |
| Pemazyre (pemigatinib)<br>2021-09-17<br>DNR | The sponsor submitted an indirect treatment comparison (ITC) of pemigatinib to a relevant comparator in Canada (FOLFOX), but there were significant limitations of the analysis, and no conclusions could be drawn regarding comparative efficacy with respect to survival outcomes (e.g., progression-free survival [PFS] and overall survival [OS]). …, but pERC was uncertain whether pemigatinib meets this need given the limitations associated with the evidence reviewed. While pERC acknowledged the rarity of FGFR2 positive CCA, and the unmet need for mor | |
| Lumakras (sotorasib)<br>2021-10-22<br>DNR | The study was open-label and the method for ascertaining PFS or OS was not reported, so the potential for and extent of any bias in the measurement of this outcome was unknown. Although the results were comparable to the PFS efficacy results reported in the CodeBreaK 100 and CodeBreaK 200 trials, the magnitude of the treatment effect | |

| | | |
|---|---|---|
| | for the real-world PFS should be interpreted with uncertainty in light of the aforementioned limitations. | |
| Jemperli (dostarlimab)<br>2021-12-23<br>DNR | There were important differences in the design of the comparator studies that limit the ability to draw strong conclusions about the effectiveness for dostarlimab compared with other treatments. An important limitation of all analyses was the fact that MMR and MSI-H status was unknown for all or most patients in the comparator trial, and it is therefore uncertain whether the comparator population in the ITC analyses would be eligible for treatment with dostarlimab, providing further uncertainty about the comparative effectiveness. | |
| Rybrevant (amivantamab)<br>2022-03-20<br>RC | A submitted adjusted indirect treatment comparison that compared amivantamab versus physician's choice of treatment and individual treatment classes (e.g., tyrosine kinase inhibitor [TKI], immunotherapy, nonplatinum chemotherapy [NPBC]) had numerous limitations and pERC concluded that no firm conclusions could be drawn on the relative efficacy of amivantamab versus relevant comparators | |
| Carvykti (citacabtagene autoleucel)<br>2023-02-09<br>RC | | Furthermore, despite uncertainty in the results due to methodological limitations (e.g., heterogeneity, risk of bias from residual confounding, small sample sizes, and imprecision), *there was consistency in the direction of effects of 5 observational studies, which favoured ciltacabtagene autoleucel over real-world treatment paradigms across all outcomes assessed, including ORR, OS, PFS,* and time to next treatment (TTNT). |
| Columvi (glofitamab)<br>2023-03-24<br>RC | Overall, the limitations of the sponsor-submitted ITCs, particularly the MAIC, including the differences in study design, included patient populations and heterogeneity in baseline characteristics across studies, as well as the reduction in sample sizes, leads to uncertainty about the overall generalizability of the results to the population living in Canada. Additionally, wide 95% CIs led to imprecision and uncertainty in the results | |
| Tecvayli (teclistamab)<br>2023-07-26<br>RC | | Furthermore, *despite uncertainty in the results of the indirect treatment comparisons (ITCs) because of methodological limitations, there was consistency in the direction of effects that favoured teclistamab over real-world physician's choice (RWPC) therapy* across the outcomes assessed, including clinical responses, OS, and PFS. |

| | | |
|---|---|---|
| Epkinly (epcoritamab)<br>2023-10-13<br>TLR | Overall, CADTH conclude that there were multiple limitations of the sponsor-submitted MAICs, including differences in inclusion and exclusion criteria, heterogeneity in baseline characteristics across studies, as well as notable reductions in sample sizes due to matching and weighting, there was significant uncertainty about the overall generalizability of the results to the Canadian population. Additionally, wide 95% CIs led to imprecision and uncertainty in the results. | |
| Elrexfio (elranatamab)<br>2023-12-06<br>RC | | *Despite uncertainty in the results of the indirect treatment comparisons and real-world evidence (RWE) cohort studies due to methodological limitations, there was consistency in the direction of effects for PFS, OS*, and complete response rate favouring elranatamab over real-world physician's choice of treatment in patients without prior exposure to BCMA-directed therapy. |

RC: Reimbursement with Conditions; DNR: Do Not Reimburse; TLR: Time Limited Reimbursement.
